# Supplementary material for: Evaluation of Health Promotion in International Schools Using the Schools for Health in Europe (SHE) Rapid Assessment Tool
Source: Healthcare (Basel). 2025 Mar 14;13(6):633. doi: 10.3390/healthcare13060633 (PMC11942499; doi:10.3390/healthcare13060633)
Supplement: Supplementary file 1 [file healthcare-13-00633-s001.zip › healthcare-3471271-supplementary file 1.pdf]

**Supplementary File 1. Schools for Health in Europe (SHE) rapid assessment tool questions**

| | Current | | | Priority | | |
|---|---|---|---|---|---|---|
| | 1 | 2 | 3 | 1 | 2 | 3 |
| 1. Orientation | | | | | | |
| 1.1 Our school has an overview of the current situation regarding pupils' health (including physical, mental and social health) and well-being. | | | | | | |
| 1.2 Our school has an overview of the current situation regarding teaching/non-teaching staffs' health (including physical, mental and social health) and well-being. | | | | | | |
| 1.3 Our school can estimate the current health behaviors (eating and physical activity, sexual activity, drinking, smoking, drugs and hygiene) of our pupils with regard to age, background and gender. | | | | | | |
| 1.4 Our school has undertaken an assessment of the needs and wishes of pupils, teaching and nonteaching staff concerning health and well- being (e.g. survey, wish boxes). | | | | | | |
| 1.5 It is known to the whole school community who is responsible for health topics in the school including mental health promotion. | | | | | | |

| | Current | | | Priority | | |
|---|---|---|---|---|---|---|
| | 1 | 2 | 3 | 1 | 2 | 3 |
| 2.Healthy school policy | | | | | | |
| 2.1 Our school has a written policy on health and wellbeing of pupils and teaching/non-teaching staff, including promoting health and well-being and preventing and dealing with health-related problems. | | | | | | |
| 2.2 Health and well-being are linked to the educational goals of our school. | | | | | | |
| 2.3 Health and well-being are part of the curriculum of our school. | | | | | | |
| 2.4 Our schools' approach to health and well-being reflects the views, wishes and needs of the whole school community (pupils, teaching/non- teaching staff and parents). | | | | | | |
| 2.5 Pupils, teaching/non-teaching staff and parents are encouraged to participate in the planning and implementation of health-related activities in the school. | | | | | | |

| | Current | | | Priority | | |
|---|---|---|---|---|---|---|
| | 1 | 2 | 3 | 1 | 2 | 3 |
| 3. School physical environment | | | | | | |
| 3.1 School facilities such as the playground, classrooms, toilets, can- teen and corridors are pupil-friendly, safe, clean and promote hygiene (enough hand soap and paper towels in the toilets) for all pupils. | | | | | | |
| 3.2 School facilities such as the playground, classrooms, toilets, canteen and corridors are appropriate with regard to age, gender and for pupils with special needs. | | | | | | |
| 3.3 Pupils and staff have access to school facilities for physical activity outside school hours. | | | | | | |
| 3.4 All the physical activity facilities and the canteen of our school meet common safety and hygiene standards. | | | | | | |
| 3.5 The route to our school is safe and designed to encourage pupils to engage in physical activity (e.g. cycling or walking). | | | | | | |
| 3.6 All of the buildings in our school are kept at a comfortable temperature, are well-lit and ventilated. | | | | | | |
| 3.7 The school canteen, the school shop and vending machines offer food and drinks that are healthy and affordable and meet national food standards. | | | | | | |

| | Current | | | Priority | | |
|---|---|---|---|---|---|---|
| | 1 | 2 | 3 | 1 | 2 | 3 |
| 4. School social environment | | | | | | |
| 4.1 Our school facilities such as canteen, playground, classrooms and corridors are designed in a pleasant and sociable way. | | | | | | |
| 4.2 Our school offers regular shared activities such as project weeks, festivals, competitions and breakfast clubs that are at least in part designed to promote health and well-being. | | | | | | |
| 4.3 Health education and health promoting activities including opportunities for practicing and developing life skills are included in after- school programmes. | | | | | | |

| | | | | | | |
|---|---|---|---|---|---|---|
| 4.4 At school a trusted person is always available for all pupils who have the need to talk with someone privately when they want to share concerns or thoughts. | | | | | | |
| 4.5 At school there is always a friendly and jovial atmosphere where all pupils and teaching/nonteaching staff feel comfortable and respected. | | | | | | |
| 4.6 School health professionals (school nurse, social worker or psychologist) are involved in individual and whole school health promotion and work together with the school management to integrate health topics into the school curriculum and policy. | | | | | | |
| 4.7 A support system (services and accommodations) is in place at our school for pupils with special learning, developmental and physical needs. | | | | | | |
| 4.8 Our school has a system for identifying and referring pupils with special needs to outside professionals if the pupil's needs are beyond the scope of the school's expertise. | | | | | | |
| | Current | | | Priority | | |
| | 1 | 2 | 3 | 1 | 2 | 3 |
| 5. Health skills | | | | | | |
| 5.1 Our school implements programs that are focused on individual skills and knowledge of health topics including mental health pro- motion. | | | | | | |
| 5.2 Our school has clear rules that promote healthy behavior. | | | | | | |
| 5.3 Activity breaks are regularly included in the school lessons and the school breaks. | | | | | | |
| | Current | | | Priority | | |
| | 1 | 2 | 3 | 1 | 2 | 3 |
| 6. Community links | | | | | | |
| 6.1 Parents of pupils in our school are active participants in the school community. | | | | | | |
| 6.2 Our school has established a connection with local partners such as sport and youth clubs, community or regional health agencies, counsel- ling services, health insurance companies, restaurants, local shops, etc. | | | | | | |

| | Current | | | Priority | | |
|---|---|---|---|---|---|---|
| 6.3 Our school arranges regular visits to local partners/stakeholders to encourage our pupils in healthy eating, physical activity, to promote their emotional or social health and development, etc. | | | | | | |
| | 1 | 2 | 3 | 1 | 2 | 3 |
| 7. Healthy school staff | | | | | | |
| 7.1 Our school offers regular teacher training and capacity building related to promoting health and well-being of the school community. | | | | | | |
| 7.2 There are sufficient resources available to provide the school staff with up-to-date materials on health topics including mental health pro- motion. | | | | | | |
| 7.3 Our school promotes a balance between work and private life, a rea- sonable workload and provides an open environment to discuss work problems and stress. | | | | | | |
| 7.4 New school staff at our school receive mentoring and training to assist them in their professional development. | | | | | | |
| 7.5 The school has a protocol for dealing with recurring staff absentee- ism and in helping returning school staff to reintegrate and adjust after a period of sick leave. | | | | | | |
| 7.6 Our school supports school staff in achieving and maintaining a healthy lifestyle, e.g., by creating a healthy enhancing environment. | | | | | | |

Table created based on information from Vilaça T, Emily D, Miranda-Velasco MJ, Martinis O, Masson J. SHE School Manual 2.0. Schools for Health in Europe Network Foundation (SHE). Haderslev, Denmark; 2020.
Available from: https://www.schoolsforhealth.org/sites/default/files/editor/health-promoting-school/she_school_manual_2.0.pdf
